# Soluble Immune-Related Proteins as New Candidate Serum Biomarkers for the Diagnosis and Progression of Lymphangioleiomyomatosis

**DOI:** 10.3389/fimmu.2022.844914

**Published:** 2022-03-01

**Authors:** Xuefei Liu, Yanping Xu, Xueying Wu, Yanpu Liu, Qiang Wu, Jialiang Wu, Henghui Zhang, Min Zhou, Jieming Qu

**Affiliations:** ^1^ Department of Pulmonary and Critical Care Medicine, Ruijin Hospital, Shanghai Jiao Tong University School of Medicine, Shanghai, China; ^2^ Institute of Respiratory Diseases, Shanghai Jiao Tong University School of Medicine, Shanghai, China; ^3^ Beijing Shijitan Hospital, Capital Medical University, Beijing, China; ^4^ Ninth School of Clinical Medicine, Peking University, Beijing, China; ^5^ School of Oncology, Capital Medical University, Beijing, China; ^6^ Department of Respiration, Xiangshan Traditional Chinese Medicine Hospital, Shanghai, China

**Keywords:** lymphangioleiomyomatosis, immune biomarkers, LAG-3, IL-18, PD-1

## Abstract

**Background:**

The goal of this study was to analyze serum from lymphangioleiomyomatosis (LAM) patients and healthy controls to identify novel biomarkers that could shed light on disease diagnosis and pathogenesis.

**Methods:**

From April 2017 to October 2019, qualified serum samples were obtained to explore differences in 59 immune proteins between 67 LAM patients and 49 healthy controls by the Luminex method.

**Results:**

We characterized 22 serum immune proteins that were differentially expressed in LAM patients compared with healthy people. Fifty-nine proteins were then classified into eight categories according to their biological function, and the results showed that LAM patients displayed significantly higher levels of growth factors (*p* = 0.006) and lower levels of costimulatory molecules (*p* = 0.008). LAG-3 was not only likely to have better predictive value than VEGF-D but also showed a significant difference between patients without elevated VEGF-D and healthy people. IL-18 was positively correlated with lung function and six-minute walk test (6MWT) distance and negatively correlated with St. George’s Respiratory Questionnaire (SGRQ) score and pulmonary artery systolic pressure (PASP), which suggested that IL-18 was related to disease severity. PD-1 was significantly different between patients with pneumothorax and/or chylothorax and those without complications.

**Conclusion:**

We performed a large-scale serum immune factor analysis of LAM. Our study provides evidence that LAG-3 may be a novel candidate serum biomarker for the diagnosis of LAM. Future independent validation in prospective studies is warranted.

## Introduction

Lymphangioleiomyomatosis (LAM) is a rare (affecting approximately five per million) and progressive cystic lung disease that occurs almost exclusively in women of reproductive age (1). LAM is characterized by excessive proliferation of atypical smooth muscle-like cells (LAM cells) and cystic destruction of the lung parenchyma. Therefore, LAM is often considered a benign tumor (2–4). LAM occurs both in sporadic cases and in patients with tuberous sclerosis complex (TSC-LAM) (5, 6). Patients can present with manifestations such as dyspnea, recurrent pneumothorax and chylothorax, renal angiomyolipomas and abdominal lymphangiomyolipomas (7). Although chest high-resolution computed tomography (HRCT) remains the preferred diagnostic method for LAM, its limitations are that LAM can be easily confused with other diseases with multiple thin-walled cysts in the lungs that similar, such as chronic obstructive pulmonary disease and cystic fibrosis. In recent years, many studies have found that excessive proliferation of LAM cells leads to lymphatic vascular malformation, which is related to the involvement of vascular endothelial growth factor D (VEGF-D) in the occurrence of the disease (8–11). Serum VEGF-D can be used as a noninvasive biomarker for the diagnosis and disease activity of LAM. However, the limitation of VEGF-D is that it is only elevated in patients with substantial lymphatic involvement, and there are still many LAM patients with non-elevated VEGF-D (12). Therefore, it is necessary to search for new serum biomarkers.

Sirolimus, a mammalian target of rapamycin (mTOR) inhibitor, is currently the only drug approved by the Food and Drug Administration (FDA) for the treatment of LAM (13–16). However, sirolimus was associated with an increased frequency of adverse events compared with placebo in the treatment of LAM (17). HHence, there is an urgent need to find novel curative targets for LAM. Recently, immunotherapy has emerged as a promising treatment for various diseases, especially tumors. As a benign tumor, preclinical data on immune checkpoint monotherapy provided further support for the use of anti-PD-1 or anti-CTLA-4 in LAM therapy (18–20), and checkpoint inhibition may be effective in parallel with rapamycin therapy (21). Large-scale exploration of LAM immunological factors can not only screen diagnostic biomarkers of LAM but may also lead to the discovery of new immunotherapeutic targets for LAM.

In our study, we collected serum samples from 67 LAM patients and 49 healthy controls and compared 59 serum immune factors measured by the Luminex method between the two groups. The goal of our study was to identify novel biomarkers that could shed light on disease diagnosis, pathogenesis and therapy.

## Methods

### Study Population and Data Collection

From April 2017 to October 2019, 67 LAM patients and 49 healthy controls at Ruijin Hospital affiliated with Shanghai Jiao Tong University were recruited for this study. All patients were diagnosed with LAM according to HRCT images showing multiple bilateral cystic shadows compatible with LAM and at least one of the following additional criteria: (i) histopathological confirmation with a biopsy; (ii) a serum VEGF-D level of ≥800 pg/ml; (iii) clinical history of chylous pleural effusion; or (iv) renal angiomyolipoma (22). All the patients were given long-term treatment of sirolimus and no one stopped taking sirolimus at the time of sampling. All the patients were in stable condition to ensure completion of the tests in this study. Written informed consent according to the Declaration of Helsinki was obtained from each patient or their legal relatives. This study was approved by the Ethics Committee of Ruijin Hospital.

### Sample Collection and Imputation

Serum samples were collected from 67 confirmed LAM patients. At the same time, we collected serum from 49 healthy people matched in age and sex as controls. Peripheral blood samples were obtained by venipuncture (10 mL, BD vacutainer blood collection tube; BD Biosciences) and centrifuged (1000 ×g, 15min) to isolate the serum. Supernatants were then stored at -80°C for later analysis. At the time of sampling, medical records on demographic characteristics and treatment were collected.

### Serum Multiplex Immunoassay

Serum samples were analyzed with a 45-plex ProcartaPlex Human Cytokine/Chemokine/Growth Factor Panel (Affymetrix, Inc.) and 14-plex ProcartaPlex Human Immuno-Oncology Checkpoint Panel (Affymetrix, Inc.). All tests were performed using a Luminex MAGPIX^®^ instrument (Luminex Co., Austin, TX, USA) according to its instructions as previously reported (23–25). ProcartaPlex Analyst 1.0 software was used for data analysis. The concentrations of cytokines (pg/mL) were determined by fitting a standard curve for mean fluorescence intensity versus concentration.

### Statistical Analysis

Quantitative variables are presented as the median ± interquartile range (IQR), and categorical variables are presented as proportions. The Mann–Whitney *U* test was used for nonnormally distributed variables. The correlations between serum proteins and different clinical indexes were analyzed by the Spearman rank correlation test. *P* values of receiver operating characteristic curve (ROC) analysis were calculated by the bootstrap method. As a summary measure of the ROC curve, the Youden index was used to determine the optimal cutoff value for the signature score. Heatmaps, the radar map, ROC curves, the correlation heatmap and boxplots were generated using R version 3.6.3 software (Institute for Statistics and Mathematics, Vienna, Austria; www.r-project.org). *P* < 0.05 was considered statistically significant, and tests were 2-tailed.

## Results

### Patient Characteristics

According to the diagnostic criteria of the European Respiratory Society (22), a total of 67 Asian female patients at a stable stage with qualified specimens were enrolled in our study. The median age was 42.00 y (IQR, 36.00 y to 49.00 y), and the median time from LAM diagnosis was 4.00 y (IQR, 2.00 y to 7.00 y). Of the 67 patients, 11.00 (16.42%) underwent surgical or bronchoscopic biopsies, 28.00 (41.8%) had pneumothorax, 28.00 (41.8%) had chylothorax, and 40.00 (59.70%) had extrapulmonary involvement, which referred to the presence of chylous ascites, angiomyolipomas and/or lymphangiomyomas of systems other than the lungs in patients with LAM. All the patients were given long-term treatment of sirolimus and in a stable stage at the time of sampling. 19.00 (28.36%) of them were treated with traditional Chinese medicine. 13.00 (19.40%) required supplemental oxygen therapy due to their poor lung function. None of the patients received antiepileptic drugs, hormonal therapy or other unspecified treatment. Previous studies reported that LAM patients often had pulmonary hypertension and decreased cardiopulmonary function (26–28), so we collected the patient’s pulmonary artery systolic pressure (PASP), six-minute walk test (6MWT), St. George’s respiratory questionnaire (SGRQ) score and lung function data at the time of sampling. The demographics and clinical characteristics of the patients are summarized in **Table 1**.

**Table 1:** Demographics and clinical characteristics of 67 LAM patients.

Characteristics	Frequency (%) or median (IQR)
Gender	
Female, n (%)	67.00 (100.00%)
Ethnic	
Asian, n (%)	67.00 (100.00%)
Age, year (IQR)	42.00 (36.00, 49.00)
Presence of TSC (%)	20.00 (29.85%)
Time from LAM diagnosis, year (IQR)	4.00 (2.00-7.00)
Biopsy, n (%)	11.00 (16.42%)
Pneumothorax, n (%)	28.00 (41.80%)
Chylothorax, n (%)	28.00 (41.80%)
Extrapulmonary involvement, n (%)	40.00 (59.70%)
Renal angiomyolipoma, n (%)	18.00 (26.87%)
Extrarenal angiomyolipoma, n (%)	9.00 (13.43%)
Lymphangioleiomyoma, n (%)	20.00 (29.85%)
Ascites, n (%)	8.00 (11.94%)
Hysteromyoma, n (%)	14.00 (20.90%)
Treatment	
Sirolimus, n (%)	67.00 (100.00%)
Traditional Chinese medicine, n (%)	19.00 (28.36%)
Supplemental oxygen, n (%)	13.00 (19.40%)
PASP, mmHg (IQR)	35.00 (31.25, 41.00)
6MWT, meter (IQR)	450.00 (360.00, 500.00)
SGRQ score (IQR)	45.63 (27.79, 62.67)
Lung function	
FEV1% predicted (IQR)	59.15 (43.03, 77.68)
FVC% predicted (IQR)	82.30 (69.62, 96.82)
FEV1/FVC (IQR)	0.77 (0.57, 0.92)
DLCO-SB% predicted (IQR)	39.10 (29.90, 56.05)

IQR, interquartile range; TSC, tuberous sclerosis; PASP, pulmonary artery systolic pressure; 6MWT, 6 minutes walk test; SGRQ, St George’ s respiratory questionnaire; FEV1, forced expiratory volume in the first second; FVC, forced vital capacity; DLCO-SB, carbon monoxide diffusing capacity-single breath.

### Comparison of Serum Immune Factors Between LAM Patients and Healthy Controls

**Figure 1:**
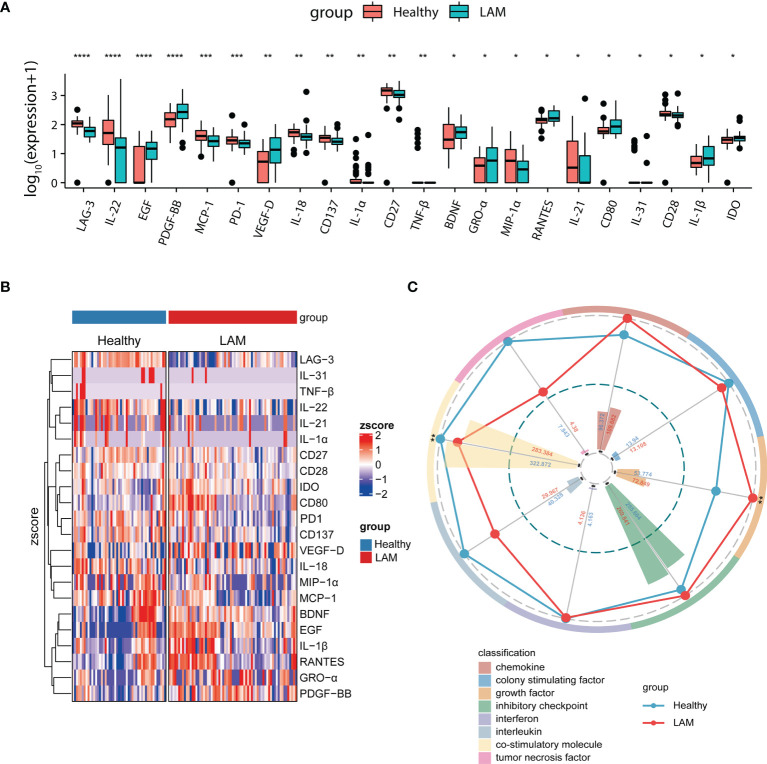
Comparison of serum immune factors between LAM patients and healthy controls. **(A)** Twenty-two differentially expressed serum proteins in LAM patients (n=67) and healthy controls (n=49). **(B)** Heatmap depicting the relative concentrations of 22 differentially expressed serum proteins between the two groups (LAM=67, Healthy=49). Each column of the heatmap shows a sample, while the rows represent different serum proteins. The color scale in the heatmap represents scores standardized across rows. **(C)** Radar map of 8 classification scores in the LAM group (n=67) compared with the healthy control group (n=49). The radius is the percentage of expression; the histograms are representative of classification scores. ****, ***, ** and * indicate p<0.0001, p<0.001, p<0.01 and p<0.05, respectively. LAM, lymphangioleiomyomatosis.

In the present study, we quantified 59 immunological factors in patient and control sera to discover LAM-related systemic changes and potential therapeutic targets. A heatmap of 59 serum factors from 67 LAM patients is shown in **Figure S1**. We classified 59 immune factors into eight categories according to their biological functions and compared their differences between LAM patients and healthy people (**Figure S2**). The levels of 22 factors in serum were significantly different between the two groups, including increased VEGF-D, IDO, CD80 BDNF, EGF, IL-1β, RANTES, GRO-α, and PDGF-BB and decreased LAG-3, IL-18, PD-1, IL-31, TNF-β, IL-22, IL-21, IL-1α, CD27, CD28, CD137, MIP-1α, and MCP-1 in LAM (all *p* < 0.05; **Figure 1A** and **Figure S2**). The heatmap in **Figure 1B** presents the 22 differentially expressed serum immune factors in LAM compared with healthy subjects.

To depict the overall differences, we then acquired the classification scores of the eight categories by computing the weighted mean expression level of the immune factors in each classification. The radar map revealed that LAM patients displayed significantly higher levels of growth factors (*p* = 0.006), as expected. Most interestingly, however, the serum level of costimulatory molecules was markedly decreased in LAM (*p* = 0.008; **Figure 1C**).

### Identification of Potential Predictive Biomarkers in LAM

**Figure 2:**
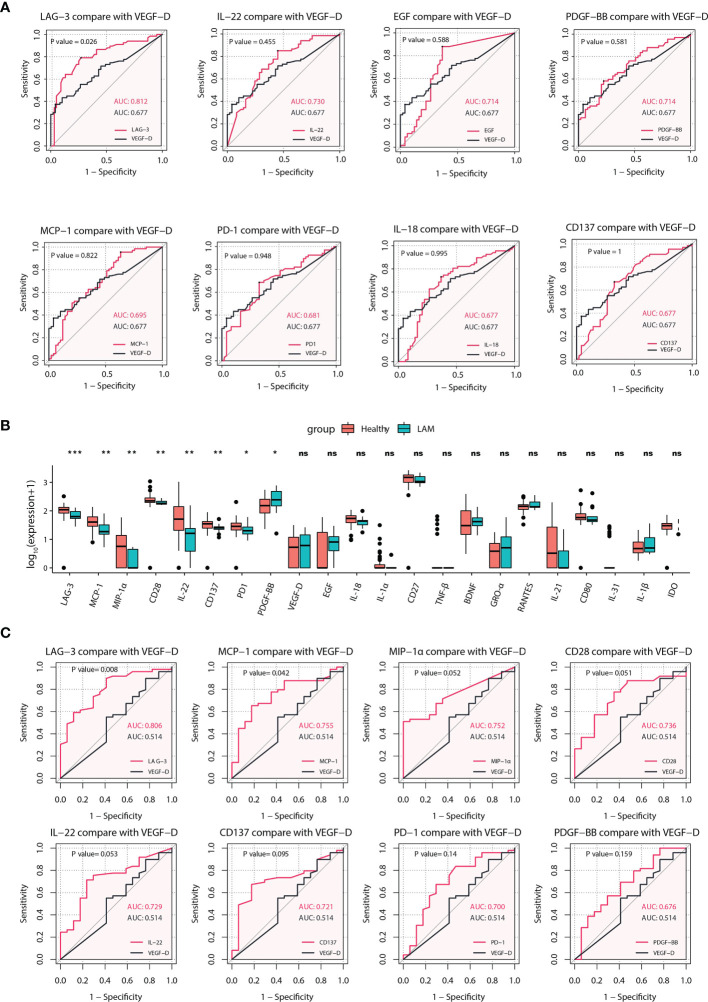
Identification of potential predictive biomarkers of LAM. **(A)** ROC analyses for LAG-3, IL-22, EGF, PDGF-BB, MCP-1, PD-1, IL-18 and CD137 with AUCs greater than or equal to that of VEGF-D (n=67). LAG-3 had the best effect, and the difference between LAG-3 and VEGF-D was statistically significant. **(B)** Comparison of 22 differentially expressed serum proteins between LAM patients whose serum VEGF-D was <800 pg/ml (n=17) and healthy subjects (n=49). Significant differences in eight serum proteins were found between the two groups. **(C)** ROC curves comparing LAG-3, MCP-1, MIP-1α, CD28, IL-22, CD137, PD-1, and PDGF-BB with VEGF-D in patients whose VEGF-D was <800 pg/ml (n=17) and healthy controls (n=49). The *p* values of the ROC curves and boxplots were calculated by the bootstrap method and Mann–Whitney *U* test, respectively. *p<0.05, **p<0.01, ***p<0.001. Values with no significant correlation are marked with “n.s.” LAM, lymphangioleiomyomatosis; ROC, receiver operating characteristic; AUC, area under curve.

VEGF-D is a widely confirmed serum biomarker for the diagnosis of LAM (8–11). However, some women with biopsy-proven LAM lack elevated VEGF-D. Therefore, it is necessary to search for biomarkers with better predictive value than VEGF-D in LAM. As shown in **Figure 2A**, the ROC curves of 59 serum immune factors were drawn to identify patients vs. healthy persons, and the area under the curve (AUC) values of LAG-3 (AUC = 0.812), IL-22 (AUC = 0.730), EGF (AUC = 0.714), PDGF−BB (AUC = 0.714), MCP-1 (AUC = 0.695), PD-1 (AUC = 0.681), IL-18 (AUC = 0.677) and CD137 (AUC = 0.677) were greater than or equal to that of VEGF-D (AUC = 0.677). In addition, comparing the ROC curve performance though the bootstrap method, it was found that the predictive effect of LAG-3 was significantly better than that of VEGF-D (*p* = 0.026). The optimal cutoff value for diagnosis corresponding to the Youden index was 82.715 pg/ml.

Subsequently, we found that 17 women in this study had VEGF-D levels in the nondiagnostic range (<800 pg/ml). To explore other biomarkers that were elevated or decreased in this subset, we compared the levels of 22 differentially expressed serum immune factors between them and healthy subjects. Interestingly, the serum levels of LAG-3 (*p* < 0.001), MCP-1 (*p* = 0.002), MIP-1α (*p* = 0.002), CD28 (*p* = 0.004), IL-22 (*p* = 0.005), CD137 (*p* = 0.007), PD-1 (*p* = 0.015) and PDGF-BB (*p* = 0.032) in this subgroup were significantly different from those in healthy subjects (**Figure 2B**), with AUC values of 0.806, 0.755, 0.752, 0.736, 0.729, 0.721, 0.700, and 0.676, respectively. Moreover, comparing the ROC curve performance though the bootstrap method, it was found that the predictive effects of LAG-3 (*p* = 0.008) and MCP-1 (*p* = 0.042) were significantly better than that of VEGF-D (**Figure 2C**). Overall, these results suggested a reasonable proxy of LAG-3 in the diagnosis of LAM, especially in patients lacking elevated VEGF-D.

### Association Between Serum Immune Factors and Clinical Severity

**Figure 3:**
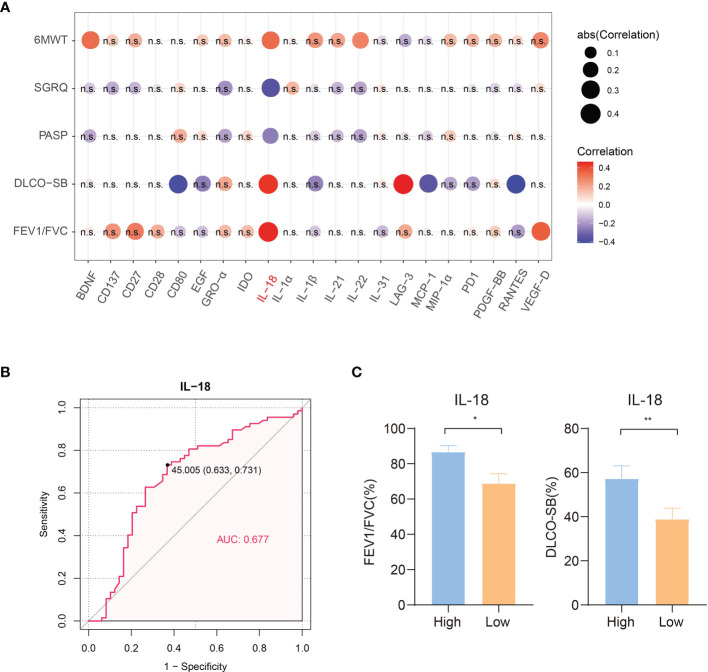
Correlation of clinical indicators and concentrations of differentially expressed proteins in 59 LAM serum samples. **(A)** Correlation heatmap of clinical indexes and concentrations of 21 differentially expressed serum proteins (TNF−beta expression was not detected in LAM patients, n=59). Red circles: positive correlation; blue circles: negative correlation. IL-18 was positively correlated with lung function and the 6MWT distance in LAM patients and negatively correlated with the PASP and SGRQ scores, which suggested that IL-18 may play a positive role in the disease process of LAM. The circle size is proportional to the absolute value of correlation *r*. Values with no significant correlation are marked with “n.s.” **(B)** ROC curve of IL-18 to distinguish LAM patients and healthy subjects (n=59). **(C)** Significantly reduced FEV1/FVC and DLCO-SB were observed for the low IL-18 expression group compared with the high IL-18 expression group (n=59). The cutoff point between high and low levels was determined using the Youden index of IL-18 (<45.005 pg/ml). Correlation *r* values were obtained using Spearman’s correlation test. *P* values were calculated by the Mann–Whitney *U* test. *p<0.05, **p<0.01. LAM, lymphangioleiomyomatosis; PASP, pulmonary artery systolic pressure; 6MWT, 6 minutes walk test; SGRQ, St George’ s respiratory questionnaire; FEV1, forced expiratory volume in the first second; FVC, forced vital capacity; DLCO-SB, carbon monoxide diffusing capacity-single breath.

To obtain insight into the correlation between serum immunological factors and disease severity to find potential targets that may predict disease severity and the quality of life of LAM patients, we collected the 6MWT distance, SGRQ score, PASP and lung function of 59 patients. A higher SGRQ score indicated a worsening of disease and a decline in quality of life. Improved 6MWT distance and lower PASP reflected better cardiopulmonary functioning. The results of correlation analyses of 21 differentially expressed serum immune factors (TNF−beta expression was not detected in LAM patients) and clinical index items are shown in **Figure 3A**. The lung diffusion capacity was positively correlated with increased serum IL-18 and LAG-3 (Spearman *r* = 0.441, *p* = 0.01; Spearman *r* =0.465, *p* = 0.006), and FEV1/FVC was positively correlated with increased serum IL-18 (Spearman r = 0.456, *p* = 0.007). The single most striking observation from the data comparison was that only serum IL-18 was negatively correlated with the SGRQ score and PASP (Spearman r = -0.354, *p* = 0.006; Spearman r = -0.255, *p* = 0.045) and had a highly positive correlation with the 6MWT distance (Spearman r = 0.336, *p* = 0.014; **Figure 3A**). Taken together, our results strongly imply that the disease severity of LAM patients was related to the level of serum IL-18. The Youden index of the ROC curve for IL-18 showed that the optimal cutoff value was 45.005 pg/ml (**Figure 3B**). Significantly reduced FEV1/FVC and DLCO-SB were observed in the low IL-18 expression group compared with the high IL-18 expression group (**Figure 3C**). The cutoff point between high and low levels was determined using the Youden index of IL-18 (greater or less than 45.005 pg/ml).

### Subgroup Analysis of LAM Patients

**Figure 4:**
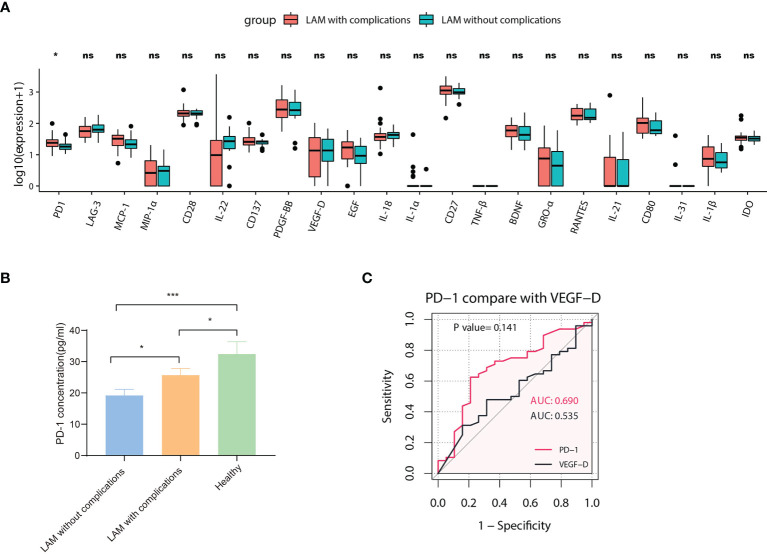
Subgroup analysis of LAM patients. **(A)** Comparison of 22 different serum proteins in LAM patients with pneumothorax or chylothorax (n=48) and LAM patients without complications (n=19). The expression of PD-1 between the two groups was statistically significant (*p*=0.015). Values with no significant correlation are marked with “ns”. **(B)** Comparison of serum PD-1 concentration among LAM patients without complications (n=19), LAM patients with complications (n=48) and healthy controls (n=49). **(C)** ROC analysis of PD-1 compared with VEGF-D in LAM patients with complications (n=48) and LAM patients without complications (n=19, *p*=0.141). Significant differences between subsets of LAM patients and healthy controls were calculated using the Mann–Whitney U test. Data are presented as the mean ± s.e.m. *p<0.05, ***p<0.001. The differences between ROC curves were measured using bootstrap analysis.

We performed a subgroup analysis based on whether LAM patients had present or prior chylothorax and pneumothorax. We compared 20 differentially expressed proteins in the serum of LAM patients without complications (n=19) and LAM patients with pneumothorax and/or chylothorax (n=48). The results showed that the level of PD-1 in the subgroup without complications was lower than that in the subgroup with complications (*p*=0.015) (**Figure 4A**). However, the levels in both subgroups were lower than those in healthy subjects (**Figure 4B**). To assess the ability of PD-1 to distinguish whether patients have complications, an ROC curve was drawn and compared with that of VEGF-D (**Figure 4C**). Although the value of PD-1 as a biomarker to identify the comorbidity of LAM was modest, the differences in PD-1 between these two subgroups and healthy individuals may indicate the underlying pathogenesis of different disease phenotypes.

### Potential Diagnostic Flowchart of LAM Combining Serum Immunological Factors

**Figure 5:**
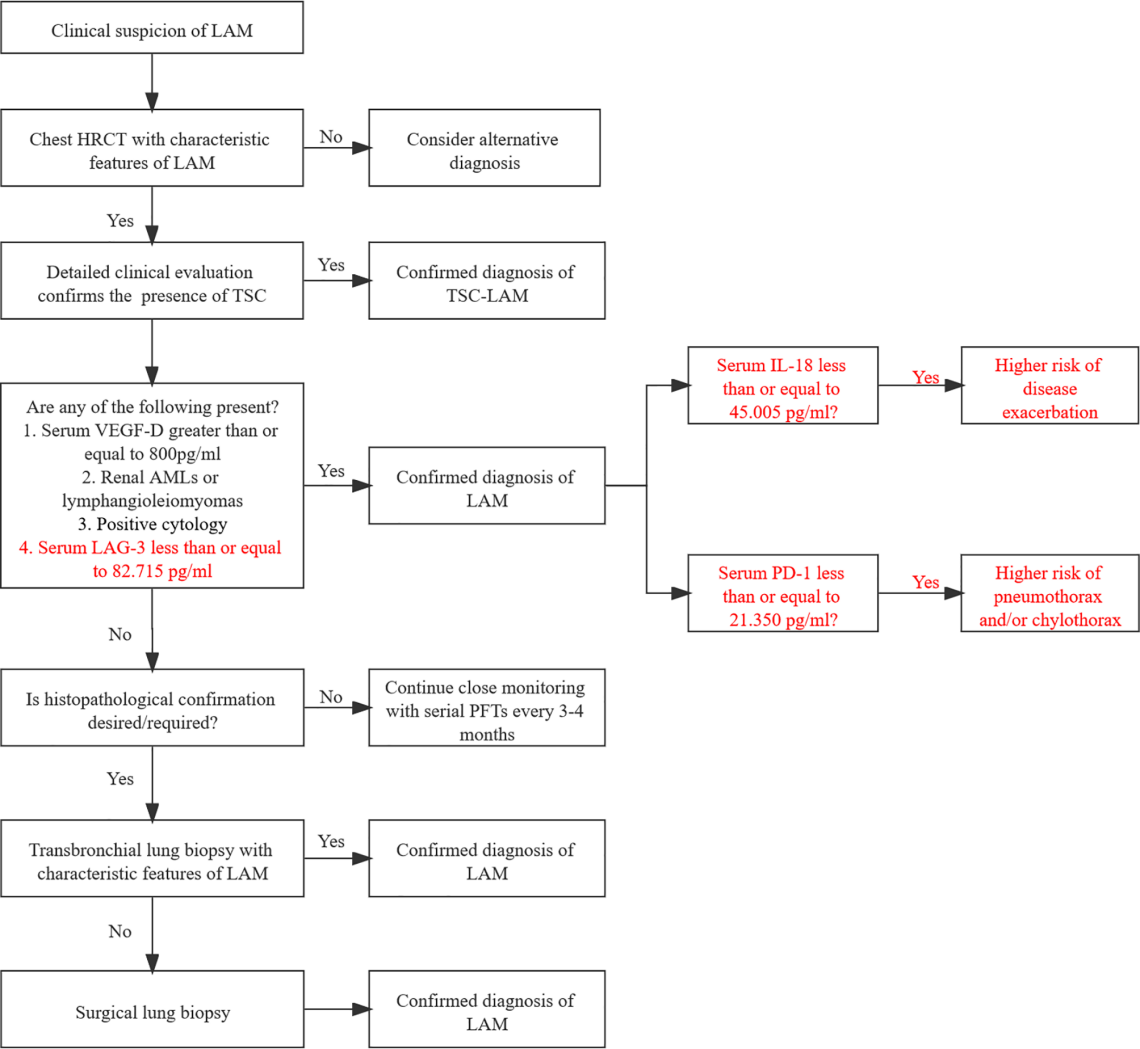
Potential diagnostic flowchart of LAM combining serum immunological factors. LAM, lymphangioleiomyomatosis; HRCT, high-resolution computed tomography; TSC, tuberous sclerosis complex; CT, computed tomography; MRI, magnetic resonance imaging; AMLs, angiomyolipoma; PFTs, pulmonary function tests.

Based on the guidelines of the American Thoracic Society/Japanese Respiratory Society (29), we established a flowchart of LAM diagnosis according to our results (**Figure 5**). The optimal cutoff value of LAG-3 for LAM diagnosis is less than or equal to 82.715 pg/ml. Patients with serum IL-18 levels less than or equal to 45.005 pg/ml may have a higher risk of disease progression, while serum PD-1 levels less than or equal to 21.350 pg/ml indicate a higher risk of pneumothorax and/or chylothorax. Although dynamic data are still warranted to validate the predictive reliability of these immune factors, they provide a direction for further independent prospective studies to identify better biomarkers than VEGF-D.

## Discussion

To our knowledge, we are the first to depict the change diagram of serum proteins, especially immune-related factors, in a relatively large cohort for this rare disease. Compared with healthy controls, we identified various immune factors in addition to the generally acknowledged VEGF-D that were differentially expressed, suggesting a potential immune function disorder in LAM patients. We found that serum LAG-3 was a better diagnostic biomarker than VEGF-D, and lower serum IL-18 was associated with more severe disease. The level of serum PD-1 was correlated with the presence of chylothorax and pneumothorax in LAM patients. These results highlighted the diagnostic value of these soluble immune-related factors and unveiled potential biological mechanisms in LAM development.

In clinic, many studies have confirmed the role of VEGF-D in the diagnosis, differential diagnosis, evaluation of disease progression and treatment of LAM (9, 17, 25). Nonetheless, there are still many LAM patients with non-elevated serum VEGF-D. On the basis of our results, serum LAG-3 was not only likely to have better predictive value than VEGF-D but also showed a significant difference between patients without elevated VEGF-D and healthy people. LAG-3 is a type I transmembrane protein similar in structure to CD4. Accumulating evidence indicates that LAG-3 is an inhibitory coreceptor that plays pivotal roles in autoimmunity, tumor immunity, and anti-infection immunity (30). As an inhibitory immune checkpoint, LAG-3, similar to PD-1, negatively regulates T cell proliferation, activation and homeostasis. Soluble LAG-3 (sLAG-3) in serum is produced by alternative splicing or cleaved by metalloproteinases from membrane-bound LAG-3 (30, 31). Although the function of sLAG-3 remains unclear, current studies suggest that, unlike membrane-bound LAG-3, sLAG-3 can induce dendritic cell activation and maturation, stimulate T cell proliferation and promote the Th1 cell response (32–35). Our results showed significantly low expression of LAG-3 in the serum of LAM patients, which we suspected may indicate inhibition of helper T cell activity. This finding is consistent with that of He et al., who found that the levels of sLAG-3 in stage III-IV non-small-cell lung cancer (NSCLC) patients were significantly lower than those in stage I-II patients, and lower sLAG-3 may be related to worse tumor immune response in advanced disease patients (36). In addition, another study showed that sLAG-3 could serve as a prognostic factor in human breast cancer expressing estrogen or progesterone receptors and that a high level of sLAG-3 predicted better overall survival (OS) (37). Thus, sLAG-3 is a better biomarker than VEGF-D in terms of diagnosis, and increasing sLAG-3 levels might be a promising treatment strategy in LAM patients. However, the utility of sLAG-3 as an early prognostic biomarker requires further confirmation in independent prospective studies.

In this study, it is somewhat surprising that the level of serum IL-18 was positively associated with better pulmonary function and 6MWT and inversely correlated with the SGRQ score and PASP in LAM. Patients with higher levels of IL-18 had better lung function. This intriguing result suggested that high serum IL-18 levels may be a protective factor in delaying the progression of LAM. Notably, recent studies highlighted the role of IL-18 in accelerating antitumor immune responses and the potential of the IL-18 pathway for immunotherapeutic intervention (38, 39). These results further support the idea that using IL-18 or activating the IL-18 pathway may serve as a potential treatment option for LAM.

In our study, it is worth noting that although PD-1 did not perform as well as LAG-3 in predicting LAM, it showed significant downregulation in LAM patients and played an important role in identifying the subgroup without increased serum VEGF-D and the subgroup with comorbidities. Recent studies found that T cells within LAM nodules and renal angiomyolipoma exhibited features of T cell exhaustion, with coinhibitory receptor PD-1 expression on tumor-infiltrating T cells. Treatment of animal models of LAM with anti-PD-1 antibodies resulted in suppression of TSC2-null tumor growth and induction of tumor rejection (18–20). These studies showed the promise of anti-PD-1 immunotherapy in LAM treatment. Soluble PD-1 (sPD-1) is bioactive and blocks the three interactions of PD-L1:B7-1, PD-L1:PD-1 and PD-L2:PD-1 to restore T cell function and proliferation and enhance immune-mediated tumor control (40–46). Similar to low expression of sLAG-3, downregulation of sPD-1 in LAM may also reflect the inhibition of T cell activity in LAM patients. A study of NSCLC patients showed that an increase in sPD-1 during erlotinib treatment was associated with prolonged progression-free survival (PFS) and OS. Improved tumor immunity mediated by increased concentrations of sPD-1 seems to play a significant role in prolonged PFS and OS in specific patients with advanced EGFR-mutated NSCLC (47). However, the immune function of sPD-1 may vary by disease, which warrants further research.

In addition to VEGF-D, we also found that LAM patients highly expressed other growth factors, such as PDGF-BB and EGF. Consistent with our results, Elena Lesma et al. found that the proliferation of LAM/TSC cells was EGF-dependent and that blockade of the EGF receptor led to cell death (48). The role of the EGFR pathway in the LAM mouse model has also been reported in previous studies by Elena Lesma et al. LAM/TSC cells in mice formed nodules expressing EGFR. Anti-EGFR antibody reduced the number and dimension of lung nodules, likely due to the inhibition of ERK and S6 signaling, reversed pulmonary alterations and reduced lymphatic and blood vessels (49). Moreover, Elena A Goncharova reported that cells dissociated from LAM nodules from the lungs of five different patients with LAM had constitutively activated S6K1, hyperphosphorylated ribosomal protein S6, activated ERK, and increased DNA synthesis compared with normal cells from the same patients. These effects were augmented by PDGF stimulation (50). Another study showed that PDGF promoted the expression of IL-13 in pulmonary smooth muscle cells through an oxidative signaling mechanism (51). These studies have preliminarily revealed the role of PDGF and EGF in LAM, and further clarification of these overexpressed growth factors and the probable underlying mechanisms is warranted. This hypothesis needs to be confirmed by further studies in the future.

Pacheco-Rodriguez G et al. found that significantly higher concentrations of CCL2 (MCP-1), CXCL1 (GRO1), and CXCL5 (ENA-78) were seen in LAM patients compared to healthy volunteers in bronchoalveolar lavage fluid (BALF) (52), which was different from the results in our paper. However, serum was used in this study instead of the BALF, different specimen types and populations may lead to different results of studies. It is worth mentioning that the levels of immune proteins in the lungs are not consistent with those in the periphery. Cui et al. showed that sirolimus treatment of 27 LAM patients reduced both VEGF-D levels and MCP-1 serum levels (53). But they did not compare the levels of MCP-1 between LAM patients and healthy people. As a rare disease, the relatively small cohort of LAM may contribute to the discrepancies between our results and other studies.

This study has several limitations. First, designing a validation cohort to confirm the findings in this study in new patients before treatment was difficult due to the rarity of the disease and the limited number of patient samples available for study. Second, it is necessary to continue dynamic follow-up of these patients to observe the changes in their serum immune factors as the disease progresses. Third, the differential diagnostic value of serum factors was not explored in cohorts of other cystic pulmonary diseases that are difficult to differentiate from LAM. These limitations will be our follow-up research direction.

In conclusion, our study described the differences in 59 immune-related factors between LAM and healthy subjects in a relatively large cohort and found that LAG-3 and IL-18 were likely biomarkers that can be used to diagnose and predict disease progression, respectively. In addition, PD-1 was found to be related to pneumothorax and chylothorax in LAM patients. The study may shed light on the diagnosis, pathogenesis and treatment of LAM. Future studies may apply these markers to test their predictive value for treatment outcome and to attempt immunotherapy targeting these factors in LAM.

## Data Availability Statement

The raw data supporting the conclusions of this article will be made available by the authors, without undue reservation.

## Ethics Statement

The studies involving human participants were reviewed and approved by Ethics Committee of Ruijin Hospital. The patients/participants provided their written informed consent to participate in this study.

## Author Contributions

JQ, MZ, and HZ designed the study. XL drafted the manuscript. XL and YX collected patients’ serum samples and recorded clinical data. XW processed statistical data. YL, QW, and JW were responsible for recruiting patients. All authors contributed to the article and approved the submitted version.

## Conflict of Interest

The authors declare that the research was conducted in the absence of any commercial or financial relationships that could be construed as a potential conflict of interest.

## Publisher’s Note

All claims expressed in this article are solely those of the authors and do not necessarily represent those of their affiliated organizations, or those of the publisher, the editors and the reviewers. Any product that may be evaluated in this article, or claim that may be made by its manufacturer, is not guaranteed or endorsed by the publisher.

## References

[1] McCormack FX Gupta N Finlay GR Young LR Taveira-DaSilva AM Glasgow CG. Official American Thoracic Society/Japanese Respiratory Society Clinical Practice Guidelines: Lymphangioleiomyomatosis Diagnosis and Management. Am J Respir Crit Care Med (2016) 194(6):748–61. doi: 10.1164/rccm.201607-1384ST PMC5803656 27628078

[2] Lynn E O'Callaghan M Sahadevan A McCarthy C. Emerging Approaches to Predict Prognosis and Monitor Disease Progression in Lymphangioleiomyomatosis. Am J Respir Crit Care Med (2019) 200(11):1424–6. doi: 10.1164/rccm.201906-1209RR 31618058

[3] Ryu JH Moss J Beck GJ Lee JC Brown KK Chapman JT. The NHLBI Lymphangioleiomyomatosis Registry: Characteristics of 230 Patients at Enrollment. Am J Respir Crit Care Med (2006) 173(1):105–11. doi: 10.1164/rccm.200409-1298OC PMC2662978 16210669

[4] McCormack FX. Lymphangioleiomyomatosis: A Clinical Update. Chest (2008) 133(2):507–16. doi: 10.1378/chest.07-0898 18252917

[5] Costello LC Hartman TE Ryu JH. High Frequency of Pulmonary Lymphangioleiomyomatosis in Women With Tuberous Sclerosis Complex. Mayo Clin Proc (2000) 75(6):591–4. doi: 10.4065/75.6.591 10852420

[6] Hancock E Tomkins S Sampson J Osborne J. Lymphangioleiomyomatosis and Tuberous Sclerosis. Respir Med (2002) 96(1):7–13. doi: 10.1053/rmed.2001.1206 11863212

[7] McCormack FX Travis WD Colby TV Henske EP Moss J. Lymphangioleiomyomatosis: Calling It What It Is: A Low-Grade, Destructive, Metastasizing Neoplasm. Am J Respir Crit Care Med (2012) 186(12):1210–2. doi: 10.1164/rccm.201205-0848OE PMC3622443 23250499

[8] Taveira-DaSilva AM Jones AM Julien-Williams P Stylianou M Moss J. Long-Term Effect of Sirolimus on Serum Vascular Endothelial Growth Factor D Levels in Patients With Lymphangioleiomyomatosis. Chest (2018) 153(1):124–32. doi: 10.1016/j.chest.2017.05.012 PMC5812752 28533049

[9] Young L Lee HS Inoue Y Moss J Singer LG Strange C. Serum VEGF-D a Concentration as a Biomarker of Lymphangioleiomyomatosis Severity and Treatment Response: A Prospective Analysis of the Multicenter International Lymphangioleiomyomatosis Efficacy of Sirolimus (MILES) Trial. Lancet Respir Med (2013) 1(6):445–52. doi: 10.1016/S2213-2600(13)70090-0 PMC3804556 24159565

[10] Young LR Vandyke R Gulleman PM Inoue Y Brown KK Schmidt LS. Serum Vascular Endothelial Growth Factor-D Prospectively Distinguishes Lymphangioleiomyomatosis From Other Diseases. Chest (2010) 138(3):674–81. doi: 10.1378/chest.10-0573 PMC2940071 20382711

[11] Young LR Inoue Y McCormack FX. Diagnostic Potential of Serum VEGF-D for Lymphangioleiomyomatosis. N Engl J Med (2008) 358(2):199–200. doi: 10.1056/NEJMc0707517 18184970 PMC3804557

[12] Banville N Burgess JK Jaffar J Tjin G Richeldi L Cerri S. A Quantitative Proteomic Approach to Identify Significantly Altered Protein Networks in the Serum of Patients With Lymphangioleiomyomatosis (LAM). PloS One (2014) 9(8):e105365. doi: 10.1371/journal.pone.0105365 25133674 PMC4136818

[13] Franz DN Krueger DA. mTOR Inhibitor Therapy as a Disease Modifying Therapy for Tuberous Sclerosis Complex. Am J Med Genet C Semin Med Genet (2018) 178(3):365–73. doi: 10.1002/ajmg.c.31655 30307123

[14] Hu S Wu X Xu W Tian X Yang Y Wang ST. Long-Term Efficacy and Safety of Sirolimus Therapy in Patients With Lymphangioleiomyomatosis. Orphanet J Rare Dis (2019) 14(1):206. doi: 10.1186/s13023-019-1178-2 31429781 PMC6702727

[15] Ando K Kurihara M Kataoka H Ueyama M Togo S Sato T. Efficacy and Safety of Low-Dose Sirolimus for Treatment of Lymphangioleiomyomatosis. Respir Investig (2013) 51(3):175–83. doi: 10.1016/j.resinv.2013.03.002 23978644

[16] Burger CD. Efficacy and Safety of Sirolimus in Lymphangioleiomyomatosis. N Engl J Med (2011) 365(3):271–2; author reply 272. doi: 10.1056/NEJMc1106358 21774717

[17] McCormack FX Inoue Y Moss J Singer LG Strange C Nakata K. Efficacy and Safety of Sirolimus in Lymphangioleiomyomatosis. N Engl J Med (2011) 364(17):1595–606. doi: 10.1056/NEJMoa1100391 PMC3118601 21410393

[18] Maisel K Merrilees MJ Atochina-Vasserman EN Lian L Obraztsova K Rue R. Immune Checkpoint Ligand PD-L1 Is Upregulated in Pulmonary Lymphangioleiomyomatosis. Am J Respir Cell Mol Biol (2018) 59(6):723–32. doi: 10.1165/rcmb.2018-0123OC PMC6293078 30095976

[19] Liu HJ Lizotte PH Du H Speranza MC Lam HC Vaughan S. TSC2-Deficient Tumors Have Evidence of T Cell Exhaustion and Respond to Anti-PD-1/Anti-CTLA-4 Immunotherapy. JCI Insight (2018) 3(8). doi: 10.1172/jci.insight.98674 PMC5931128 29669930

[20] LiuHJKrymskayaVPHenskeEP. Immunotherapy for Lymphangioleiomyomatosis and Tuberous Sclerosis: Progress and Future Directions. Chest (2019) 156(6):1062–7. doi: 10.1016/j.chest.2019.08.005 31437431

[21] Pluvy J Brosseau S Stelianides S Danel C Nguenang M Khalil A. Safe and Effective Use of Nivolumab for Treating Lung Adenocarcinoma Associated With Sporadic Lymphangioleiomyomatosis: A Rare Case Report. BMC Pulm Med (2019) 19(1):12. doi: 10.1186/s12890-018-0775-5 30634951 PMC6329093

[22] Johnson SR Cordier JF Lazor R Cottin V Costabel U Harari S. European Respiratory Society Guidelines for the Diagnosis and Management of Lymphangioleiomyomatosis. Eur Respir J (2010) 35(1):14–26. doi: 10.1183/09031936.00076209 20044458

[23] Ji S Chen H Yang K Zhang G Mao B Hu Y. Peripheral Cytokine Levels as Predictive Biomarkers of Benefit From Immune Checkpoint Inhibitors in Cancer Therapy. BioMed Pharmacother (2020) 129:110457. doi: 10.1016/j.biopha.2020.110457 32887027

[24] Lu Z Zou J Hu Y Li S Zhou T Gong J. Serological Markers Associated With Response to Immune Checkpoint Blockade in Metastatic Gastrointestinal Tract Cancer. JAMA Netw Open (2019) 2(7):e197621. doi: 10.1001/jamanetworkopen.2019.7621 31339548 PMC6659353

[25] Zhao C Wu L Liang D Chen H Ji S Zhang G. Identification of Immune Checkpoint and Cytokine Signatures Associated With the Response to Immune Checkpoint Blockade in Gastrointestinal Cancers. Cancer Immunol Immunother (2021) 70(9):2669–79. doi: 10.1007/s00262-021-02878-8 PMC10992426 33624146

[26] Cottin V Harari S Humbert M Mal H Dorfmuller P Jais X. Pulmonary Hypertension in Lymphangioleiomyomatosis: Characteristics in 20 Patients. Eur Respir J (2012) 40(3):630–40. doi: 10.1183/09031936.00093111 22362861

[27] Freitas CSG Baldi BG Jardim C Araujo MS Sobral JB Heiden GI. Pulmonary Hypertension in Lymphangioleiomyomatosis: Prevalence, Severity and the Role of Carbon Monoxide Diffusion Capacity as a Screening Method. Orphanet J Rare Dis (2017) 12(1):74. doi: 10.1186/s13023-017-0626-0 28427470 PMC5399314

[28] Wu X Xu W Wang J Tian X Tian Z Xu K. Clinical Characteristics in Lymphangioleiomyomatosis-Related Pulmonary Hypertension: An Observation on 50 Patients. Front Med (2019) 13(2):259–66. doi: 10.1007/s11684-018-0634-z 29675687

[29] Gupta N Finlay GA Kotloff RM Strange C Wilson KC Young LR. Lymphangioleiomyomatosis Diagnosis and Management: High-Resolution Chest Computed Tomography, Transbronchial Lung Biopsy, and Pleural Disease Management. An Official American Thoracic Society/Japanese Respiratory Society Clinical Practice Guideline. Am J Respir Crit Care Med (2017) 196(10):1337–48. doi: 10.1164/rccm.201709-1965ST PMC5694834 29140122

[30] Maruhashi T Sugiura D Okazaki IM Okazaki T. LAG-3: From Molecular Functions to Clinical Applications. J Immunother Cancer (2020) 8(2). doi: 10.1136/jitc-2020-001014 PMC7488795 32929051

[31] Anderson AC Joller N Kuchroo VK. Lag-3, Tim-3, and TIGIT: Co-Inhibitory Receptors With Specialized Functions in Immune Regulation. Immunity (2016) 44(5):989–1004. doi: 10.1016/j.immuni.2016.05.001 27192565 PMC4942846

[32] El Mir S Triebel F. A Soluble Lymphocyte Activation Gene-3 Molecule Used as a Vaccine Adjuvant Elicits Greater Humoral and Cellular Immune Responses to Both Particulate and Soluble Antigens. J Immunol (2000) 164(11):5583–9. doi: 10.4049/jimmunol.164.11.5583 10820232

[33] Buisson S Triebel F. MHC Class II Engagement by its Ligand LAG-3 (CD223) Leads to a Distinct Pattern of Chemokine and Chemokine Receptor Expression by Human Dendritic Cells. Vaccine (2003) 21(9-10):862–8. doi: 10.1016/S0264-410X(02)00533-9 12547595

[34] Triebel F. LAG-3: A Regulator of T-Cell and DC Responses and its Use in Therapeutic Vaccination. Trends Immunol (2003) 24(12):619–22. doi: 10.1016/j.it.2003.10.001 14644131

[35] Annunziato F Manetti R Tomasevic I Guidizi MG Biagiotti R Gianno V. Expression and Release of LAG-3-Encoded Protein by Human CD4+ T Cells are Associated With IFN-Gamma Production. FASEB J (1996) 10(7):769–76. doi: 10.1096/fasebj.10.7.8635694 8635694

[36] He Y Wang Y Zhao S Zhao C Zhou C Hirsch FR. sLAG-3 in non-Small-Cell Lung Cancer Patients' Serum. Onco Targets Ther (2018) 11:4781–4. doi: 10.2147/OTT.S164178 PMC6097502 30147330

[37] Triebel F Hacene K Pichon MF. A Soluble Lymphocyte Activation Gene-3 (sLAG-3) Protein as a Prognostic Factor in Human Breast Cancer Expressing Estrogen or Progesterone Receptors. Cancer Lett (2006) 235(1):147–53. doi: 10.1016/j.canlet.2005.04.015 15946792

[38] Ma Z Li W Yoshiya S Xu Y Hata M El-Darawish Y. Augmentation of Immune Checkpoint Cancer Immunotherapy With IL18. Clin Cancer Res (2016) 22(12):2969–80. doi: 10.1158/1078-0432.CCR-15-1655 26755531

[39] Zhou T Damsky W Weizman OE McGeary MK Hartmann KP Rosen CE. IL-18BP is a Secreted Immune Checkpoint and Barrier to IL-18 Immunotherapy. Nature (2020) 583(7817):609–14. doi: 10.1038/s41586-020-2422-6 PMC7381364 32581358

[40] Zhu X Lang J. Soluble PD-1 and PD-L1: Predictive and Prognostic Significance in Cancer. Oncotarget (2017) 8(57):97671–82. doi: 10.18632/oncotarget.18311 PMC5722594 29228642

[41] He L Zhang G He Y Zhu H Zhang H Feng Z. Blockade of B7-H1 With sPD-1 Improves Immunity Against Murine Hepatocarcinoma. Anticancer Res (2005) 25(5):3309–13. 16101143

[42] He YF Zhang GM Wang XH Zhang H Yuan Y Li D. Blocking Programmed Death-1 Ligand-PD-1 Interactions by Local Gene Therapy Results in Enhancement of Antitumor Effect of Secondary Lymphoid Tissue Chemokine. J Immunol (2004) 173(8):4919–28. doi: 10.4049/jimmunol.173.8.4919 15470033

[43] Liu C Jiang J Gao L Wang X Hu X Wu M. Soluble PD-1 Aggravates Progression of Collagen-Induced Arthritis Through Th1 and Th17 Pathways. Arthritis Res Ther (2015) 17:340. doi: 10.1186/s13075-015-0859-z 26608464 PMC4659197

[44] Onlamoon N Rogers K Mayne AE Pattanapanyasat K Mori K Villinger F. Soluble PD-1 Rescues the Proliferative Response of Simian Immunodeficiency Virus-Specific CD4 and CD8 T Cells During Chronic Infection. Immunology (2008) 124(2):277–93. doi: 10.1111/j.1365-2567.2007.02766.x PMC2566632 18266718

[45] Shin SP Seo HH Shin JH Park HB Lim DP Eom HS. Adenovirus Expressing Both Thymidine Kinase and Soluble PD1 Enhances Antitumor Immunity by Strengthening CD8 T-Cell Response. Mol Ther (2013) 21(3):688–95. doi: 10.1038/mt.2012.252 PMC3589170 23337984

[46] Song MY Park SH Nam HJ Choi DH Sung YC. Enhancement of Vaccine-Induced Primary and Memory CD8(+) T-Cell Responses by Soluble PD-1. J Immunother (2011) 34(3):297–306. doi: 10.1097/CJI.0b013e318210ed0e 21389868

[47] Sorensen SF Demuth C Weber B Sorensen BS Meldgaard P. Increase in Soluble PD-1 Is Associated With Prolonged Survival in Patients With Advanced EGFR-Mutated Non-Small Cell Lung Cancer Treated With Erlotinib. Lung Cancer (2016) 100:77–84. doi: 10.1016/j.lungcan.2016.08.001 27597284

[48] Lesma E Ancona S Sirchia SM Orpianesi E Grande V Colapietro P. TSC2 Epigenetic Defect in Primary LAM Cells. Evidence of an Anchorage-Independent Survival. J Cell Mol Med (2014) 18(5):766–79. doi: 10.1111/jcmm.12237 PMC4119383 24606538

[49] Lesma E Chiaramonte E Ancona S Orpianesi E Di Giulio AM Gorio A. Anti-EGFR Antibody Reduces Lung Nodules by Inhibition of EGFR-Pathway in a Model of Lymphangioleiomyomatosis. BioMed Res Int (2015) 2015:315240. doi: 10.1155/2015/315240 25699271 PMC4324894

[50] Goncharova EA Goncharov DA Spaits M Noonan DJ Talovskaya E Eszterhas A. Abnormal Growth of Smooth Muscle-Like Cells in Lymphangioleiomyomatosis: Role for Tumor Suppressor TSC2. Am J Respir Cell Mol Biol (2006) 34(5):561–72. doi: 10.1165/rcmb.2005-0300OC PMC2644221 16424383

[51] Bansal G Wong CM Liu L Suzuki YJ. Oxidant Signaling for Interleukin-13 Gene Expression in Lung Smooth Muscle Cells. Free Radic Biol Med (2012) 52(9):1552–9. doi: 10.1016/j.freeradbiomed.2012.02.023 PMC3341529 22370092

[52] Pacheco-Rodriguez G Kumaki F Steagall WK Zhang Y Ikeda Y Ikeda P. Chemokine-Enhanced Chemotaxis of Lymphangioleiomyomatosis Cells With Mutations in the Tumor Suppressor TSC2 Gene. J Immunol (2009) 182(3):1270–7. doi: 10.4049/jimmunol.182.3.1270 PMC2947111 19155472

[53] Cui Y Steagall WK Lamattina AM Pacheco-Rodriguez G Stylianou M Kidambi P. Aberrant SYK Kinase Signaling Is Essential for Tumorigenesis Induced by TSC2 Inactivation. Cancer Res (2017) 77(6):1492–502. doi: 10.1158/0008-5472.CAN-16-2755 PMC5358967 28202529

