# Supplementary material for: Soluble Immune-Related Proteins as New Candidate Serum Biomarkers for the Diagnosis and Progression of Lymphangioleiomyomatosis
Source: Front Immunol. 2022 Mar 1;13:844914. doi: 10.3389/fimmu.2022.844914 (PMC8923288; doi:10.3389/fimmu.2022.844914)

**Supplementary Materials for**

**Soluble immune-related proteins as new candidate serum biomarkers for the diagnosis and progression of lymphangioleiomyomatosis**

Xuefei Liu^1,2#^, Yanping Xu^1,2#^, Xueying Wu^3#^, Yanpu Liu^4^, Qiang Wu^4^, Jialiang Wu^4^, Henghui Zhang^3*^, Min Zhou^1,2*^ and Jieming Qu^1,2*^

**Figure S1. Heatmap of 59 serum immune factors in 67 LAM patients.** Each column of the heatmap shows a sample, while the rows represent different serum proteins.

**
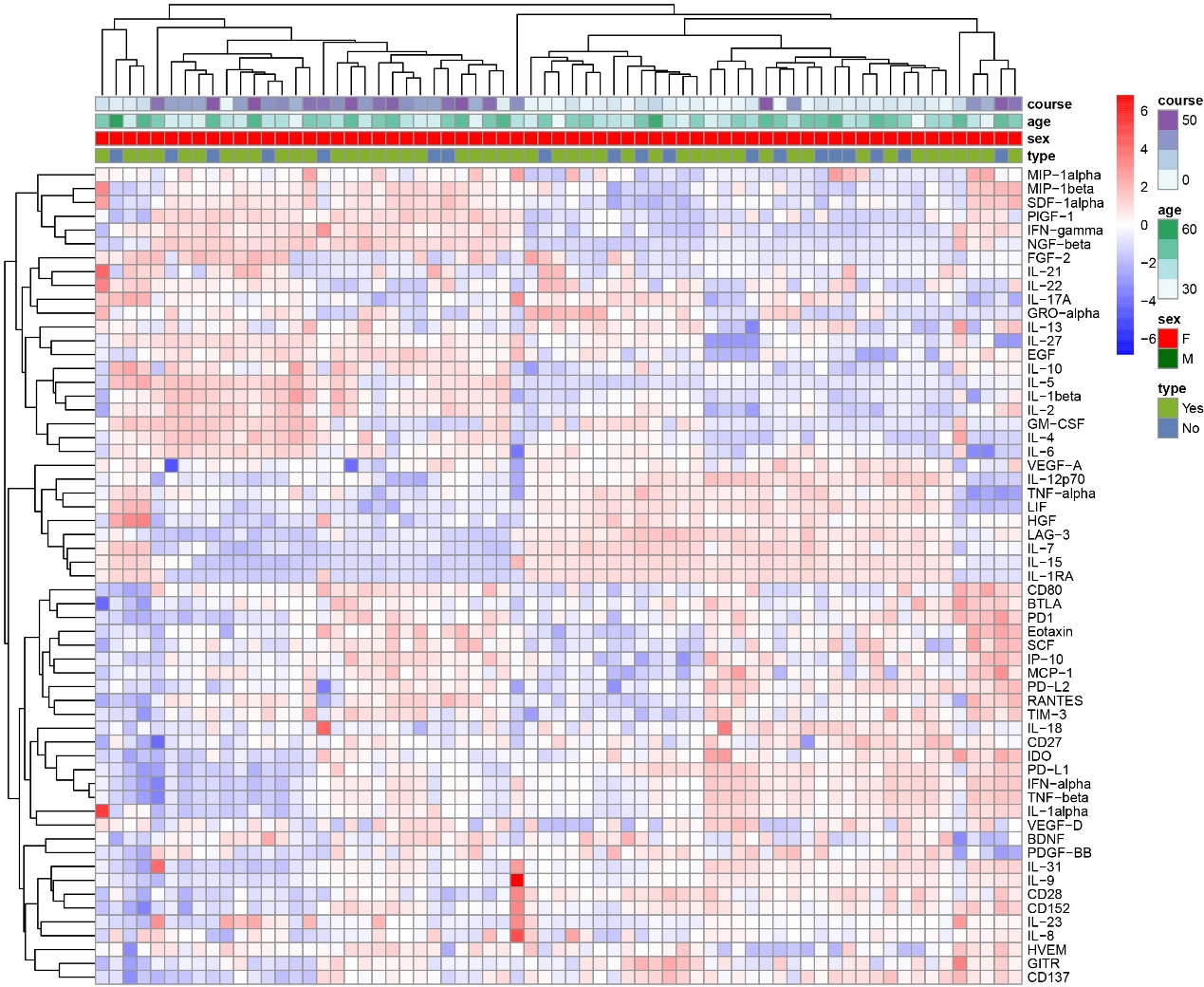
**

**Figure S2. Comparison of 59 serum immune factors between LAM patients and healthy subjects.** Fifty-nine serum immune factors can be divided into eight categories according to their types and functions. **p*<0.05, ***p*<0.01, ****p*<0.001.


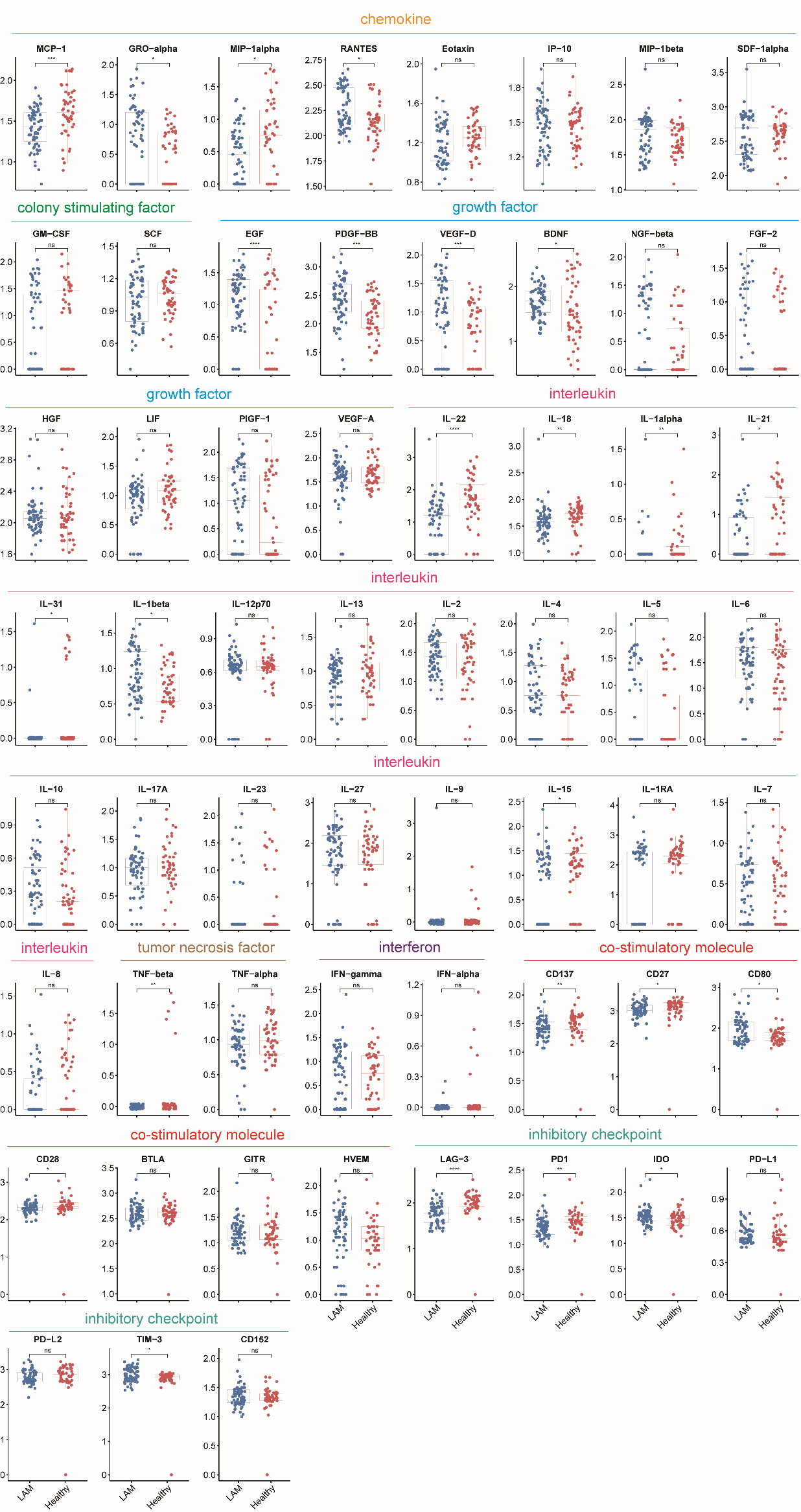

Supplement: Supplementary file 1 [file DataSheet_1.docx]
